# Microwave Breast Imaging System Modules, Enhancing Scan Quality and Reliability of Diagnostic Outputs During Clinical Testing

**DOI:** 10.3390/bioengineering12101079

**Published:** 2025-10-03

**Authors:** Giannis Papatrechas, Angie Fasoula, Petros Arvanitis, Luc Duchesne, Alexis Raveneau, Julio Daniel Gil Cano, John O’ Donnell, Sami Abd Elwahab, Michael Kerin

**Affiliations:** 1 Wavelia Healthcare, MVG Industries, 10563 Athens, Greece; 2 Wavelia Healthcare, MVG Industries, 91140 Villejust, France; 3 Discipline of Surgery, Lambe Institute for Translational Research, School of Medicine, University of Galway, H91TK33 Galway, Ireland; 4 Department of Surgery, Galway University Hospital, Saolta University Healthcare Group, H91YR71 Galway, Ireland

**Keywords:** microwave imaging, breast imaging, breast surface reconstruction, medical radar, breast cancer diagnosis

## Abstract

Microwave Breast Imaging (MWBI) is an emerging imaging modality aiming to detect breast lesions, which are dielectrically contrasted against the background healthy tissue, in the microwave frequency spectrum. MWBI holds potential to outperform X-ray mammography’s low sensitivity in young and dense breasts, thus supporting timelier detection of interval cancers, as a supplemental screening or diagnostic imaging method. The specificity of MWBI remains unknown, however, as management of false positives has not been systematically addressed yet. An earlier First-In-Human clinical investigation on 24 symptomatic patients provided proof-of-concept for the Wavelia MWBI sectorized multi-static radar imaging technology, which generates clinically meaningful 3D images of the breast, performs semi-automated detection of breast lesions and extracts diagnostic features to distinguish malignant from benign lesions. This paper focuses on a set of technological upgrades, accessories and data processing modules, designed and implemented in the 2nd generation prototype of Wavelia, to handle the diversity in breast geometry, tissue consistency and deformability, in a larger clinical investigation reporting on the bilateral MWBI scan of 62 patients. The presented add-on modules contribute to enhanced quality of scan and a more valid reference reporting space for the MWBI imaging outputs, with a direct positive impact on overall specificity.

## 1. Introduction

Microwave Breast Imaging (MWBI) [1,2,3,4,5,6,7,8,9] employs electromagnetic waves of very low power in the microwave frequency spectrum [0.5–9] GHz, to detect and localize the presence of dielectrically contrasted breast tissues, on which the emitted waves are scattered before reception by the scanner. Malignant and denser breast tissues are associated with higher permittivity values compared to the average normal breast tissue [10,11,12,13] and are expected to generate radar echoes of non-negligible intensity, associated with their inherent dielectric contrast with the underlying breast parenchyma. The dielectric contrast, a priori increased in tissues with higher concentrations of water [14,15], is a physical property not exploited yet in state-of-the-art diagnostic breast imaging [16].

Despite the numerous studies and system prototypes having performed clinical tests with various distinct implementations of the MWBI technology by now [17,18,19,20,21,22,23,24,25,26,27,28], MWBI remains an active research field, requiring further investigation, to understand its full potential and identify the specific clinical cases in which it may bring significant added value, as a supplemental screening or diagnostic imaging modality.

The Wavelia MWBI system employs sectorized multi-static radar imaging technology, enabling 3D volumetric imaging of the breast [29,30,31,32], semi-automated detection of breast lesions based on intensity and persistence, and generation of radar signatures associated with the histological classification of the breast lesions [33]. In a First-In-Human (FiH) study on 24 symptomatic patients (NCT03475992) [34], which was conducted at the Clinical Research Facility of Galway (CRFG), Ireland, the Wavelia#1 prototype showed potential to detect and characterize breast lesions based on shape and texture descriptors of Regions-Of-Interest (ROIs), extracted from MWBI-based 3D volumetric breast images [35,36]; the majority of the lesions (21 out of the 24) were detectable, and the patients reported overall positive feedback of the MWBI examination procedure.

To our knowledge, Wavelia is the only MWBI prototype system that generates 3D volumetric images of the breast for visual inspection and clinical interpretation, in conjunction with a comprehensive image analysis set per extracted ROI, to support semi-automated detection and characterization of breast lesions.

Based on the experience gained from the FiH clinical investigation with Wavelia#1, an upgraded prototype (Wavelia#2) was manufactured and recently tested in a pilot clinical investigation including a larger and more diverse dataset of 62 patients, attending the Symptomatic Breast Unit of University Hospital Galway, Ireland (NCT05757427) [37]. The technical upgrade of the system prototype Wavelia#2 was intended to ensure enhanced performance and a better level of stability for the MWBI scan. The achieved quality and repeatability of the MWBI scan were technically assessed on complex anthropomorphic breast phantoms [38], after installation of the Wavelia#2 prototype at the clinical investigation site and prior to initiation of the pilot clinical investigation [32].

Apart from the fundamental axes of technical upgrade of Wavelia#2, which were earlier presented in [32] and revisited again in Section 2.1, in this article, a set of additional modules, accessories and tailored data processing tools is presented. They were designed and implemented in Wavelia#2 MWBI to handle the diversity in the geometry and tissue consistency of real breasts, in a larger pilot clinical investigation compared to the FiH study. The diversity of the Wavelia#2 clinical investigation dataset is highlighted in Section 2.2 in the Materials and Methods section. The following Section 2.3, Section 2.4 and Section 2.5 are dedicated to the additional new modules of Wavelia#2, which are associated with direct interaction between the human body and the MWBI scanner and were not part of the experimental validation of the Wavelia#2 system with anthropomorphic breast phantoms, as earlier presented in [32]. These were benchmarked during the exploratory pilot clinical investigation with Wavelia#2 instead and are first presented in this paper. They include:

- Ergonomic accessories, which contribute to improved immersion of the breast in the transition liquid and deeper ultimate insertion in the scanning zone.
- An optical system embedded in the MWBI scanner and contributing to: (a) significant improvement in breast positioning in the scanner, (b) better management of the achieved quality of scan, and (c) rationalization of the ROI localization in the MWBI images and its association to pre-diagnosed lesions in reference imaging data, in cases of misaligned, non-vertical and/or twisted insertion of the breast in the MWBI scanner. This new optical system was introduced in the pilot clinical investigation with Wavelia#2, while the Optical Breast Contour Detection (OBCD) subsystem that was implemented in a separate examination table and formed part of the Wavelia#1 scanner, as presented in detail in [39] and employed during the FiH clinical investigation, was rendered obsolete for Wavelia#2.
- A new data processing module that was designed and implemented during the pilot clinical investigation with Wavelia#2, to assess the quality of the MWBI scan at the uppermost coronal section being scanned, close to the examination table, and reject portions of it, resulting in MWBI image formation and analysis based on a partial MWBI scan, if justified. The algorithm and set of criteria for scan quality assessment are based on geometrical analysis of the reconstructed contour of the breast. A dedicated module for MWBI scan-based breast contour reconstruction was part of the Wavelia data processing methodology since its early development [40], albeit significantly upgraded recently, in the scope of the Wavelia#2 development, as introduced in [41]. The importance of the breast contour reconstruction to the MWBI achieved imaging quality was earlier investigated in the MWBI research community, and various distinct methodologies have been considered and accordingly developed [42,43,44,45,46,47]. A breast contour assessment-based estimation tool, employed to define a partial MWBI scan of sufficient quality to deliver reliable images and image analysis outputs, i.e., ROIs and associated features, for meaningful clinical analysis, was, for the first time, conceived and implemented for Wavelia#2.

Where applicable, threshold values were empirically chosen in the current early phase of development of the new modules as part of the conducted exploratory investigation on a 62-patient diverse dataset. Generalizability across larger populations, breast densities, and system geometries, including threshold sensitivity analyses and study of failure modes, is pending to be implemented in future confirmatory clinical studies with subsequent versions of the MWBI scanner having reached a Technology Readiness Level (TRL) of 6 to 7 before.

## 2. Materials and Methods

### 2.1. The Wavelia#2 Microwave Breast Imaging (MWBI) Scanner Prototype

**Figure 1:**
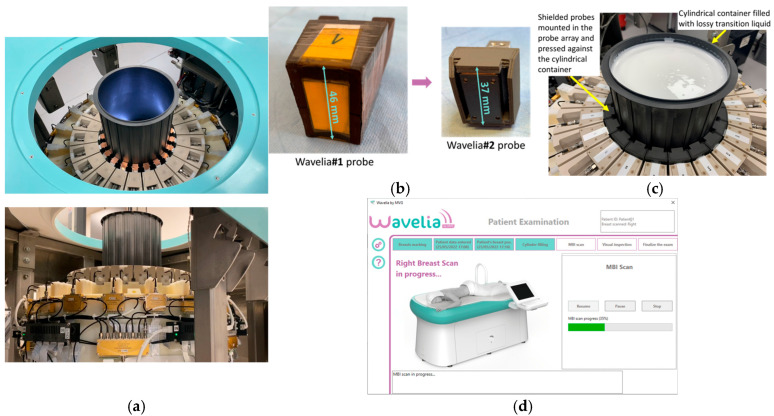
The upgraded Wavelia#2 MWBI scanner: (**a**) The multifaceted cylindrical container and the circular probe array attached to its wall to perform a cylindrical scan of the pendulous breast, while sliding vertically along the container; (**b**) The smaller dipole-like probes, embedded in dielectric material, of the Wavelia#2 system—comparison with the larger Wavelia#1 Vivaldi probes. Combined with a thinner examination table, more complete scan of the breast inserted in the scanner is achieved, approaching 13 mm closer to the surface of the examination table, compared to Wavelia#1; (**c**) The chemically stabilized creamy transition liquid in which the breast is immersed during MWBI scan and top view of the probes shielded with layers of magnetic material; (**d**) Interface of the data acquisition embedded software.

The following are the main adaptations which have been implemented in the 2nd generation prototype of the Wavelia MWBI system (developed by Wavelia Healthcare, MVG Industries, Villejust, France and Athens, Greece), as part of its fundamental technical upgrade transitioning from Wavelia#1: (a) increase of the internal radius of the cylindrical container of the scanner—from 79 mm in Wavelia#1 to 90 mm in Wavelia#2—to facilitate better scan quality for larger breasts, (b) integration of smaller antennas to improve imaging of the posterior part of the breast, (c) use of upgraded Radio-Frequency (RF) components and cabling to improve the stability of the recorded signals and enhance the sensitivity to weaker signals, (d) integration of a thermoregulation unit to enhance temperature control and improve the uniformity of the temperature inside the device over the full breast scan, (e) chemical stabilization of the transition liquid, which still remains difficult to logistically manage (7 L of transition liquid required per patient scan), (f) automation of the filling and emptying of the MWBI scanner’s dedicated cylindrical container with transition liquid, (g) integration of an optical system of endoscopic cameras within the MWBI scanner to enable aid-to-breast positioning, (h) development of an advanced interface for the data acquisition software, to guide the operator and verify adherence to the predefined examination procedure, step-by-step, (i) finally, an upgraded mechanical support was developed for the probe array, to achieve good horizontality level and adherence to the cylindrical container, while maintaining controlled inter-spacing of the probes during the full 3D scan of the breast. Photos of the upgraded modules in the Wavelia#2 MWBI scanner are shown in Figure 1.

### 2.2. Wavelia#2 Pilot Clinical Investigation

#### 2.2.1. The Study Dataset

In the Wavelia#2 clinical investigation, 73 patients were enrolled in total. 62 of them had a valid dataset of reference imaging and bilateral MWBI scan and were considered evaluable for this study. They were all symptomatic patients, presenting with a palpable breast lump > 1 cm. The study dataset was balanced, including 32 patients with cancers and 30 patients with benign lesions.

Patients were excluded from this study if they were pregnant, breastfeeding, had a an A cup size or insufficient breast volume for MWBI, breast surgery within the previous 12 months, aesthetic breast implants, non-removable or active metallic implants (excluding biopsy clips), non-intact breast skin due to bleeding, inflammation, oedema, or insufficient healing if post-biopsy, significant comorbidities posing risk, or were deemed unsuitable for scanning or unlikely to remain prone for 15–30 min.

**Figure 2:**
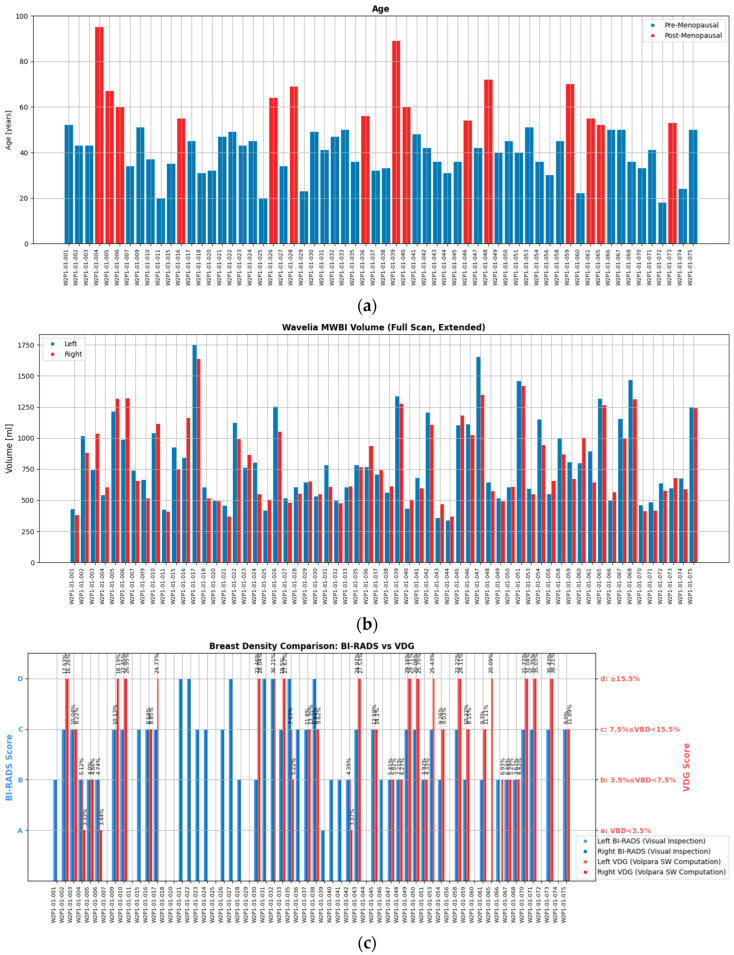
The diverse study population of the Wavelia#2 clinical investigation: (**a**) Patient age; (**b**) MWBI scanned volume; (**c**) Breast density category.

A wide range of ages, breast sizes, and breast density profiles, with a significant majority being young dense breasts, were included in the study. Distributions of the study population profile factors are shown in Figure 2, highlighting the diversity of the dataset in terms of breast geometry and background breast tissue composition, resulting in significantly varying levels of deformability of the breast in the MWBI scanner.

‘For processing’ DICOM datafiles of the mammograms were available at the clinical investigation site for 54.03% of the MBWI breast scans. This additional data allowed quantitative assessment of the Volumetric Breast Density (VBD), categorization as per Volpara Density Grade (VDG) and estimation of the volume of the scanned breast using the Volpara Lab 2.0 software (Volpara Health Technologies Ltd, Wellington, New Zealand) [48,49]. For the subset of patients for whom such mammography-based computations were possible, red bars are additionally present in Figure 2c to indicate the VDG score of the breast for direct comparison with the Breast Density Category attributed to each breast by the study radiologist, which is based on visual inspection of the patient’s mammogram as per BIRADS Atlas 5th Edition [50]. In this study, an R&D license of the Volpara Lab software, with a customized set of outputs, was used. This also allowed us to export spatial maps of dense tissue thickness computed on both standard mammographic views (Cranio-Caudal, CC and Medio-Lateral Oblique, MLO), as later depicted in Section 3.1, where the layout of a representative patient study dataset from the Wavelia#2 clinical investigation is presented.

#### 2.2.2. The Main Study Outcomes: Diagnostic Performance and Usability of the Wavelia#2 MWBI Scanner

In this clinical investigation, lesion detection and sizing were assessed relative to standard imaging techniques and histology, where applicable. The primary study outcome was breast lesion detectability, while secondary endpoints consisted of the accuracy of lesion sizing and detection rates relative to biopsy clip presence. The primary and secondary outcomes were reported with 95% confidence intervals, while also reporting subgroup analyses by patient age, breast density category and lesion size. Lesion characterization by means of the extraction of discriminating features between malignant and benign lesions was also explored, and results were reported in [51].

Stratified lesion detection results:

The Wavelia#2 MWBI system demonstrated an overall 90.3% sensitivity for lesion detection (95% CI: [80.45–95.49%]), detecting malignant and benign lesions in 84.4% and 96.7% of patients, respectively. The system demonstrated notably accurate sensitivity in dense breasts (BI-RADS C/D: 91.4% (32/35)) and smaller lesions of 10–15 mm size (100% (14/14)), and also displayed satisfactory performance in younger patients, detecting lesions in 95.5% of those under 40 years of age (21/22; 95% CI: [78.20–99.19%]) and 96.0% of pre-menopausal patients above 40 years of age (24/25; 95% CI: [80.46–99.29%]). Overall, detection rates were comparable across subgroups regardless of age, breast density, or presence of biopsy clips.

MWBI-based lesion sizing: concordance with ultrasound:

The accuracy of MWBI-based lesion sizing compared to standard-of-care imaging techniques (i.e., ultrasound) was measured by the maximal linear size difference in millimeters. Mean lesion size as measured by the Wavelia#2 System was 19.1 mm (SD = 6.3), compared to 22.6 mm (SD = 13.5) by ultrasound. The mean maximal linear size difference between modalities was −3.3 mm (SD 14.0), reflecting a modest average underestimation by Wavelia. The median size difference was −1.6 mm (range: −67.7 mm to +21.6 mm), reflecting variable agreement across the cohort. These findings suggested general concordance between modalities, albeit notable variation exhibited in individual cases.

Lesion characterization: malignant-to-benign lesion discriminating features:

MWBI Region-Of-Interest (ROI) extraction and characterization based on multidimensional radiomic feature vectors was implemented in this study to expand on the malignant-to-benign lesion diagnostics potential of MWBI, compared to the limited scope of the FiH study with Wavelia#1, which employed three specific, preselected features. Malignant-to-benign lesion separability analysis using the ANOVA F-statistic was conducted and demonstrated significant diagnostic accuracy for a set of texture and intensity-based features of the ROIs, as extracted from the Wavelia#2 MWBI 3D volumetric study images. The results of this analysis are reported in [51].

Phenomenological qualitative assessment of specificity in healthy contralateral breasts of the enrolled patients:

The malignant-to-benign lesion separability analysis was further expanded to assess the separability between malignant lesions and unspecified findings (i.e., non-clinically relevant ROIs extracted from the Wavelia MWBI images) in this study. The phenomenological preliminary assessment of specificity presented in [51] suggested a ~10% false-positive rate for Wavelia MWBI. A total of four unspecified findings of significant intensity, non-characterizable as imaging artefacts nor separable from malignant lesions, were identified in a set of 41 analyzed asymptomatic contralateral breasts. As further discussed in [51], once a sufficient maturity level of the MWBI technology is achieved to allow proper implementation of objective quantitative assessment of the MWBI specificity, validation, including comparisons against established imaging modalities, is pending to be integrated in future confirmatory studies.

**Figure 3:**
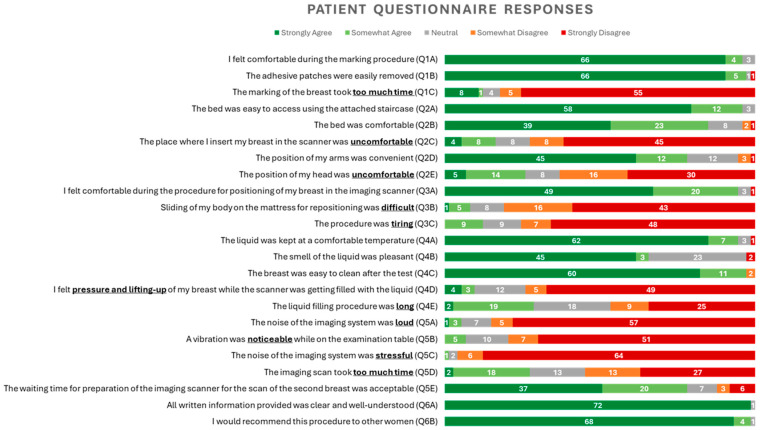
Patients’ questionnaire responses.

Patient-reported outcomes: usability and comfort of the Wavelia#2 MWBI scanner:

In Figure 3, the patients’ responses to a questionnaire are presented in the form of a cumulative bar graph. The questionnaire was completed by all 73 patients who underwent the MWBI breast examination. The questions are shown directly in front of each bar of the graph. The color code is explained in the legend of the graph, ranging from dark green (strongly agree) to dark red (totally disagree). The numbers of answers are directly inserted in each bar. It is noted that the results of the answers to the questions Q1C, Q2C, Q2E, Q3B, Q3C, Q4D, Q4E, Q5A, Q5B, Q5C, and Q5D were considered positive if the patients disagreed (dark red or orange bars).

As can be seen from the graph, the patients’ feedback was overall positive, with the exception of the following aspects, which were slightly more mixed: (a) The position of my head was uncomfortable (Q2E); (b) The liquid filling procedure was long (Q4E); (c) The imaging scan took too much time (Q5D); and (d) The waiting time for preparation of the imaging scanner for the scan of the second breast was acceptable (Q5E).

In addition to feedback to Q2E, which indicates that improvements should be considered regarding the positioning of the head, all the other mixed responses (Q4E, Q5D, and Q5E) were linked to the duration of the procedure in general. This duration is planned to be reduced in the future, which will potentially enhance clinical acceptance of the device.

The new modules presented in the sequel of this paper were designed to achieve enhanced Field-Of-View by means of deeper immersion of the breast in the MWBI scanner and a more standardized positioning of the breast, yielding images of better quality and diagnostic reliability. The patients’ feedback on usability and comfort of the device (reported here) was overall positive, highlighting the clinical feasibility of the system.

### 2.3. The Ergonomic Interface of the Wavelia#2 Examination Table with the Breast

**Figure 4:**
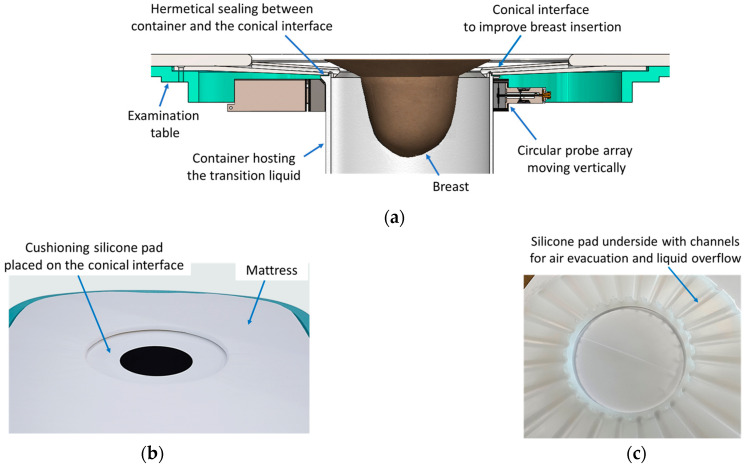
The examination table-to-breast ergonomic interface of the Wavelia#2 MWBI scanner prototype: (**a**) Detailed view of the region where the breast is placed during the MWBI scan; (**b**) Silicon pad designed to improve the comfort of the patient; (**c**) Tailored design of the cushioning silicon pad, aimed at evacuation of the air and overflowing transition liquid, thus allowing optimized immersion of the breast in the scanner.

The Wavelia#2 prototype features an enhanced table-to-breast interface, illustrated in Figure 4a,b. A conical-shaped entry design has been introduced to facilitate greater breast immersion depth. Additionally, a cushioning silicone pad, shown in Figure 4c, serves two purposes. First, its tailored design includes dedicated exhaust paths for the air and overflowing transition liquid (Figure 4c), which further enhances the achievable immersion depth. Second, it contributes to improved patient comfort during the scan.

The data presented in Figure 1 of [52], which reports on the First-In-Human (FiH) clinical study, illustrated the comparison between breast volume estimates obtained from the Wavelia OBCD scanner, X-ray mammography (via Volpara software), and the Wavelia MWBI scanner. Figure 1a of [52] served to validate the OBCD breast volume computation against the X-ray mammography breast volume data, supporting the reliability of the OBCD-derived volume computation. In contrast, Figure 1b demonstrated a noticeable discrepancy between the volumes computed from the OBCD and the MWBI scan data. This discrepancy was symptomatic of both the insufficient immersion of the breast in the scanner and the significant deformation of the breast when immersed in the transition liquid. The data presented further suggested that large breast size and advanced age are the two main factors contributing to this volume mismatch. Larger breasts tend to achieve reduced immersion depth due to anatomical constraints, while in elderly patients, breast tissue is generally less dense, making it more susceptible to deformation from the transition liquid.

Significant improvement has been achieved in the Wavelia#2 pilot clinical investigation in terms of breast immersion depth, thanks to the improved ergonomics of the examination table and the integrated aid-to-breast-positioning (multi-endoscopic system) module described in the following section. In the FiH study, we relied on the OBCD optical system to provide a reference for breast anatomy and size, compensating for variability in breast positioning and volume due to patients lying on two distinct examination tables for the OBCD and MWBI scans as discussed in Section II.B of [52]. This approach was necessary to mitigate the inherent challenges of consistent patient alignment across scans. In the current study, however, with the introduction of optimized table ergonomics and the dedicated aid-to-breast-positioning module, we no longer depend on OBCD measurements. Instead, MWBI-derived data alone suffice, as the resulting volume computations have proven significantly more reliable compared to those obtained during the FiH clinical trial.

**Figure 5:**
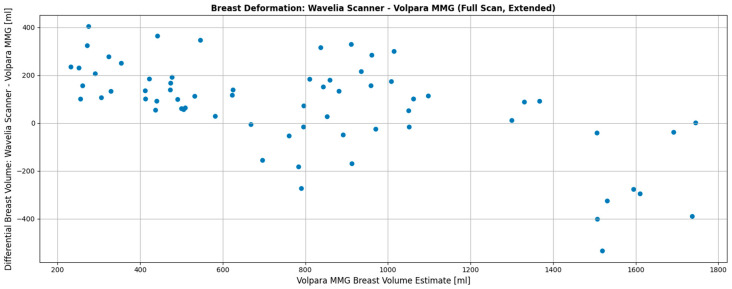
Wavelia#2 MWBI full scanned volume: comparison to breast scan volume based on X-ray mammogram (Volpara Lab estimation).

Figure 5 presents data from the Wavelia#2 clinical investigation. It can be observed that smaller breasts tend to be inserted more deeply in the Wavelia#2 scanner, occasionally enabling excess surrounding tissue to enter the scanning field, resulting in overestimation of volume in the MWBI scan. Conversely, very large breasts >1400 mL are systematically not inserted deeply enough inside the MWBI scanner to assure a scanned volume comparable with the X-ray mammography. Nevertheless, over a quite wide range of volumes for medium-sized and not excessively large breasts, the volume discrepancy between MWBI and X-ray mammography remains within acceptable limits (±200 mL), highlighting the effectiveness of the previously discussed system improvements.

### 2.4. The Aid-to-Breast Positioing Module: Endoscopic Cameras Employment for Inspection and Partial Control of the Breast Position in the Scanner

**Figure 6:**
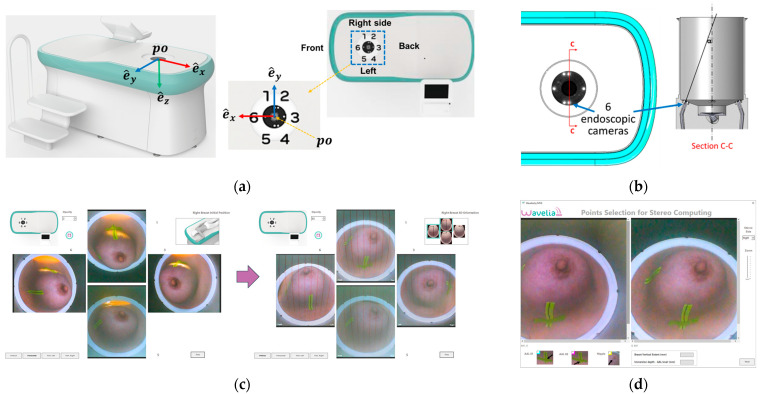
The Aid-to-breast positioning module, based on a multi-endoscopic camera system integrated in the Wavelia#2 MWBI scanner prototype: (**a**) Wavelia#2 MWBI prototype, coordinate system and endoscopic cameras configuration; (**b**) Top view of the six endoscopic cameras (left), cut view (right); (**c**) Software toolkit breast position tracking and verification display; (**d**) Software toolkit key points selection for stereo computations.

The Wavelia#2 prototype has been upgraded with the integration of an ‘aid-to-breast positioning’ module, based on a system of six endoscopic cameras located at the bottom of the scanner in the cylindrical container. The setup of these cameras is shown in Figure 6a,b. The objective is to support and enhance the proper positioning of the breast before filling the cylindrical container with the opaque creamy transition liquid, for the MWBI scan to run. A software toolkit has been developed, intended to verify and track the breast centering, the breast verticality, the breast azimuthal orientation and the potentially off-centered location of the nipple on the pendulous breast based on the endoscopic cameras’ inputs. This information is feedbacked in real-time, guiding the operator through optimization of the patient position, with examples of such visual feedback shown in Figure 6c.

**Figure 7:**
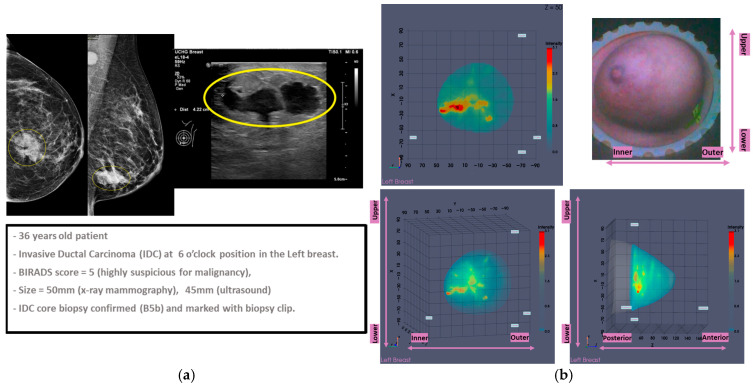
Patient 035, Left breast, IDC at 6 o’clock: Example of clinically validated ROI on the Wavelia MWBI scan, only because endoscopes were available: (**a**) Reference patient study data; (**b**) Wavelia MWBI imaging outputs.

After the breast positioning is complete, images from the six endoscopic cameras are recorded to capture the achieved state, including nipple location, azimuthal orientation, and vertical inclination of the breast. Once the MWBI scan is finalized, these images assist in valid ROI localization in the breast, enabling clinical association with findings on available reference images (X-ray mammography, ultrasound scan, or MRI). An example of an ROI validated with the aid of the endoscopic system is illustrated in Figure 7 below.

The improved breast positioning achieved through this system directly impacts the overall quality of the MWBI scan. By assisting in centered positioning and mitigation of skin folds within the imaging scene, the resulting MWBI images are less prone to positioning-induced artifacts. As a result, the likelihood of fictitious or ambiguous ROIs is reduced, supporting more reliable image interpretation and enhancing the diagnostic value of the scan.

**Figure 8:**
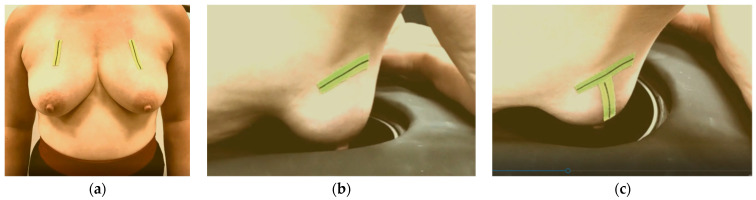
Breast marking for Wavelia#2 MWBI scan: (**a**) C-N line marking on both breasts of a volunteer at standing position; (**b**) AAL line marking on the right breast of a volunteer, while lying on the examination table of the MWBI scanner; (**c**) BP line added.

The significance of proper breast centering and alignment to assure acceptable quality of scan has also been studied in detail for the Magnetic Resonance Imaging (MRI) scanners [53]. MRI breast imaging is also performed at the prone position, and the patient setting closely resembles the one used in Wavelia MWBI, thus MRI ergonomic study outputs for image artefact mitigation and image quality enhancement can offer valuable inputs to the Wavelia developments. As part of the implemented breast marking methodology in [54], three anatomical reference lines have been defined to assist with breast alignment in the scanner (Figure 8):

1. The C-N line, connecting the midpoint of the clavicle (C) to the nipple (N), is used to control the azimuthal orientation of the breast and ensure valid quadrant definition in the MWBI images.
2. The AAL line (Anterior Axillary Line), which is used to control the immersion depth of the breast in the scanner, provides a means to better quantify the ratio of distance-to-nipple over distance-to-chest wall for the identified ROIs.
3. The BP line. This is the line perpendicular to the AAL line and passing through the nipple of the breast. The purpose of marking the BP line is to control the verticality of the breast in the scanner, for valid breast quadrant definition on the MWBI images.

These additions to the positioning process, combined with the visual guidance and verification offered by the endoscopic camera system, contribute significantly to improving both image interpretability and overall scan consistency.

### 2.5. The MWBI Breast Contour Extraction Module: Semi-Automated Definition of Partial Scan Extent

MWBI imaging relies on analyzing backscattered received signals and, therefore, it heavily depends on knowing the physical boundaries of the various propagation media along the wave path. Hence, accurate breast surface estimation is fundamental, as it defines the interface between the transition liquid and the breast. Any inaccuracies in the definition of the breast boundary can significantly degrade image quality.

Our initial approach to breast surface reconstruction, introduced in [40], was recently refined in [41], leading to more accurate single-height estimations and greater flexibility for future improvements.

Despite overall strong performance, estimating the breast contour near the chest wall remains difficult, especially in very small or too large breasts. These challenges stem from the probe array’s fixed geometry, which limits adaptability and results in cross-coupling artifacts (in cases of large breasts) or permits inclusion of the inframammary fold, the pectoral muscle and chest wall within the imaging scene (in cases of small breasts). Such inaccuracies compromise both image quality and diagnostic reliability.

To address this, two tailor-made estimation tools were developed to detect low-fidelity regions near the chest wall and generate partial scans, preserving only the reliable portion of each MWBI scan for image reconstruction and analysis.

#### 2.5.1. Partial Scan Generation for Enhanced Imaging Fidelity—Large Breasts

This section addresses challenges associated with large breast geometries. Due to their extended periphery and the buoyant force exerted by the transition liquid, large breasts often deform, bringing skin near the chest wall close to the probe array. Highly reflective dielectric surfaces, such as the skin approaching the antenna elements, increase mutual coupling between antennas, leading to strong cross-coupling where emitted energy is directly picked up by neighboring antennas. Additionally, when the skin is too close to the antennas, it can cause significant surface reflections, preventing proper wavefront propagation and masking regions deeper in the breast, where lesions could be otherwise present and detectable in proper MWBI scan conditions.

To mitigate these effects, a dedicated tool has been developed. It identifies the upper breast region in such cases, where imaging fidelity is compromised. It evaluates the distance between the estimated breast contour and the probe array of the MWBI scanner, flagging coronal slices of the pendulous breast where the skin is too close to the probe array. These regions are then excluded from subsequent imaging and analysis steps.

The detailed mathematical formulation and parameter definitions of the large breasts tool are provided in Appendix A.

**Figure 9:**
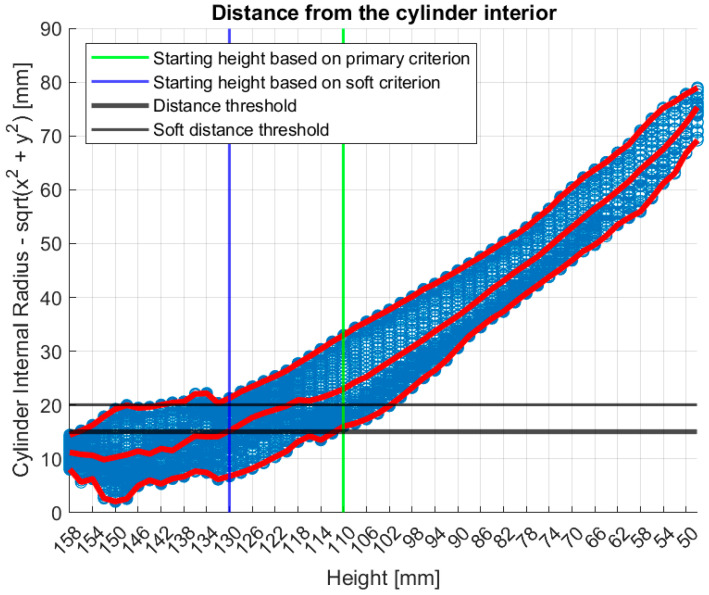
Graphical illustration of the large breasts tool’s decision process. Blue points represent the distance between each point of the breast contour estimate and the probe array, while red points represent the maximum, the mean and the minimum values of the distances at each height (i.e., vertical scan position of the probe array).

Figure 9 illustrates the tool’s functionality on Patient 017, Left breast, a representative large breast case with a volume of 1745.2 mL. In that case, the soft indicator (see Appendix A) was employed as the primary one (see definition in Appendix A),would have resulted in excessive filtering.

In Figure 9, the numerical “height” values indicate the specific vertical positions at which measurements were acquired. The uppermost scanning position of the probe array is defined as *h_uppermost_* = 158 mm, while the bed level is located at *h_bed_* = 182.5 mm. The imaging system’s z-coordinate is related to the height (*h*) by the expression $z=h_{bed}-h$, such that *z_min_* = 24.5 mm corresponds to *h_uppermost_* = 158 mm.

#### 2.5.2. Partial Scan Generation for Enhanced Imaging Fidelity—Small Breasts

This section addresses challenges related to small breast geometries. Small breasts typically immerse deeper into the scanner, often including non-breast anatomical structures such as excess chest tissue, bones, and pectoral muscle within the imaging scene. This results in distortion of the breast geometry near the chest wall, where the breast periphery appears artificially wider, altering the relative proportions of breast tissue and transition liquid. Such distortion compromises the accuracy of the assumed model and introduces uncertainty in TR-MUSIC multi-static radar imaging results [29,31]. Additionally, strong scatterers like bones and pectoral muscle are associated with high permittivity in the microwave frequency spectrum [11,15], when compared to healthy breast tissue, thus risking generating strong reflections that may mask radar echoes from weaker scatterers of interest in the breast, potentially obscuring diagnostically relevant features.

Currently, no measures have been implemented to mitigate the impact of bones or pectoral muscle inclusion in the imaging scene. However, a dedicated tool has been developed for identifying near-chest wall regions where geometry is notably disrupted by non-mammary tissue. It analyzes height-by-height the estimated breast contour areas, detecting abrupt area decreases in adjacent heights, indicative of non-breast tissue presence. Similar to the approach employed for large breast geometries, it excludes the identified regions from image formation and analysis.

A full technical description of the small breasts tool, including equations, parameter definitions and algorithmic steps, is provided in Appendix B.

**Figure 10:**
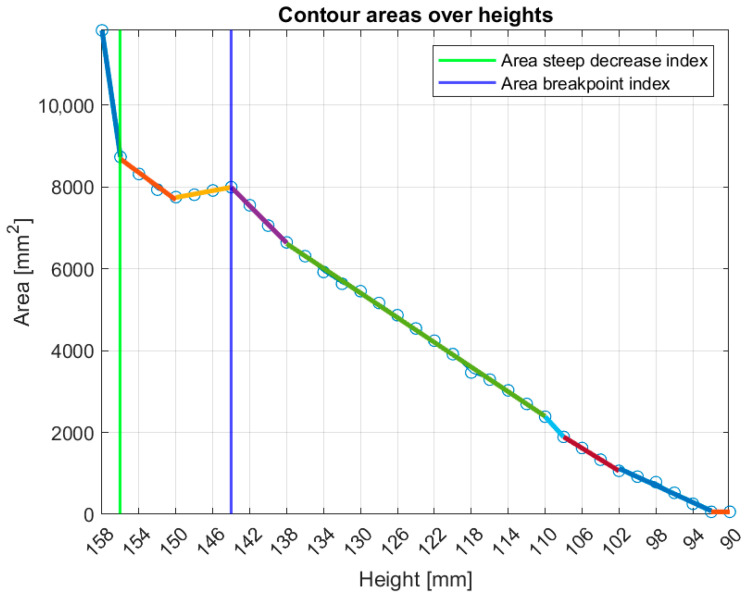
Graphical illustration of the small breasts tool’s decision process. The colored linear segments represent the piecewise linear approximation of the ***areas*** (see Appendix B) vector.

Figure 10 illustrates the tool’s functionality for Patient 048, Right breast, a representative small breast case with a volume of 509.5 mL, where the *area-breakpoint* indicator (see definition in Appendix B) was employed due to insufficient filtering of the *area-steep-decrease* indicator (see definition in Appendix B).

## 3. Results

This section presents indicative results obtained using the developed tools on patient datasets from the Wavelia#2 clinical investigation. Section 3.1 includes reference data and a complete set of imaging and image analysis outputs from the MWBI scan of a typical patient case of a small breast, to illustrate the Wavelia#2 MWBI system’s performance and underscore the significance of the developed tools. Section 3.2 examines the individual operation of each tool across different scenarios, presenting selected cases that illustrate their functionality and behavior. Finally, Section 3.3 presents an analysis of the study dataset based on breast volume, evaluating the overall impact of the tools and offering additional insights into volume trends and key patterns observed across different breast size categories.

### 3.1. MWBI Partial Scan of Acceptable Quality and Relevance: Full Example of MWBI Patient Scan Imaging and Image Analysis Outputs for Radiological and Clinical Analysis

**Figure 11:**
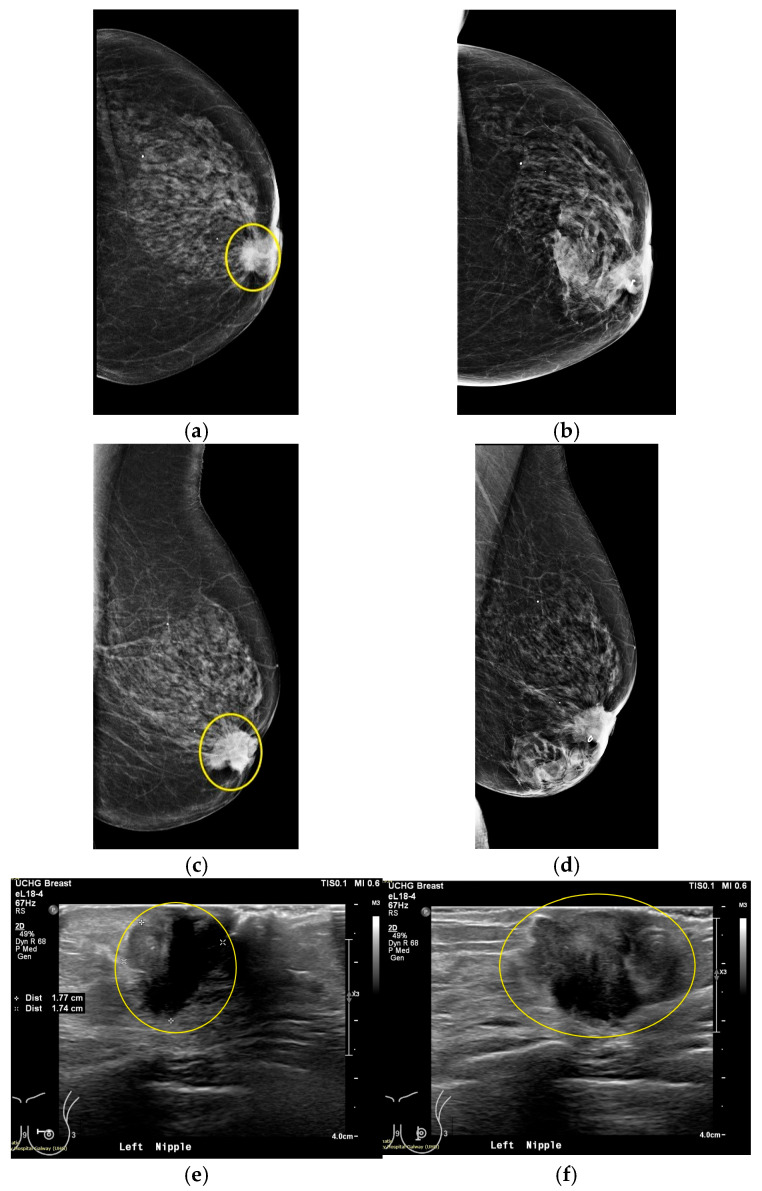
Patient 059, Left breast reference radiological data. The images correspond to: (**a**) Annotated MMG CC view, performed before core biopsy of the lesion; (**b**) post-biopsy MMG CC view; (**c**) MMG MLO view before biopsy; (**d**) post-biopsy MMG MLO view; (**e**) US first view with lesion sizing; (**f**) US second view.

This section discusses the case of Patient 059, Left breast scan. In Figure 11, the related mammogram (MMG) and ultrasound (US) images are presented, provided by the clinical investigation site as reference data for this patient study.

The lesion is clearly outlined in the mammographic views in Figure 11a,c behind the nipple, where on the post-biopsy mammographic views in Figure 11b,d, the metallic clip marking the lesion location in the breast is visible. From the US images in Figure 11e,f, the location of the lesion is indicated at 9 o’clock (upper inner quadrant for MMG reference) alongside its dimension, which is estimated to be 20 mm on Ultrasound scan, and 22 mm on X-ray mammography, based on radiological reports provided by the hospital.

Subsequently, the information retrieved by the Volpara Lab [48,49] software analysis on the pre-biopsy MMGs is presented.

**Figure 12:**
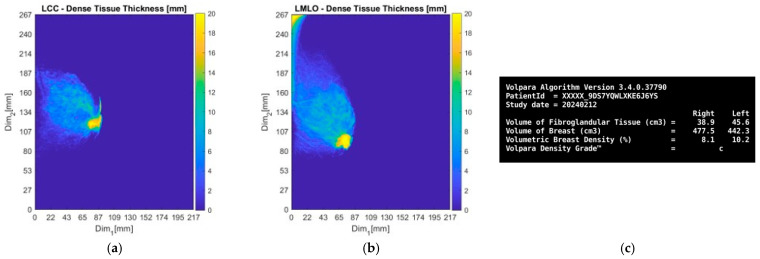
Patient 059, Left breast: selected Volpara Lab software outputs. The images correspond to: (**a**) CC view dense tissue thickness map; (**b**) MLO view dense tissue thickness map; (**c**) Volumetric Breast Density (VBD) computational outputs summary.

From the dense tissue thickness maps in Figure 12a,b (customized outputs exported from Volpara Lab for the purpose of the study), it is seen that the lesion region is way denser than the rest of the breast, being easily detectable and localized by visual inspection of the mammogram. In terms of breast density grade, Volpara assigned the score of C, while breast density category B was qualitatively assigned by the study radiologist’s visual inspection, as per BIRADS Atlas 5th Edition (2013) [50], indicating a moderately dense breast overall.

**Figure 13:**
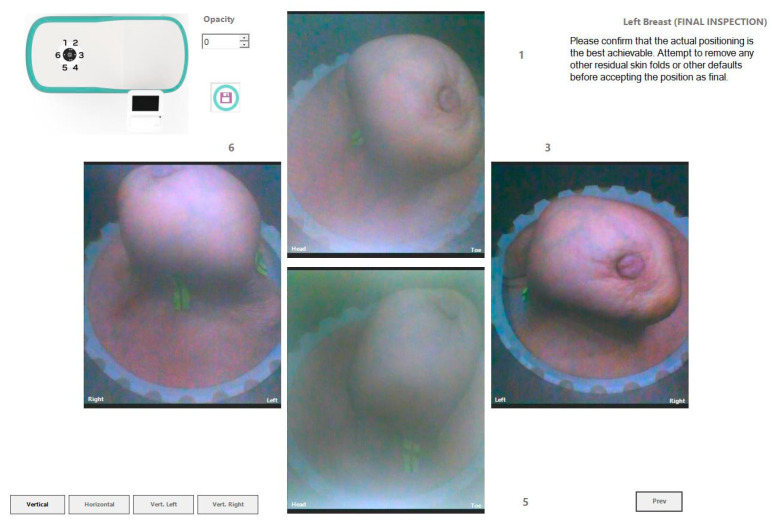
Patient 059, Left breast, Views recorded by the four endoscopic cameras: 1 (Right side), 3 (Lower side), 5 (Left Side), 6 (Upper side).

In Figure 13, the endoscopic camera views of the breast inside the Wavelia MWBI scanner are presented.

From Figure 13, it is observed that the patient’s breast has been sub-optimally positioned in the MWBI scanner, with excess tissue entering the scanner, and so an artificially widened reconstructed breast surface is expected near the chest. It is also seen that the breast is significantly distorted, especially in close vicinity to the nipple, where the cancerous lesion is known to be located.

**Figure 14:**
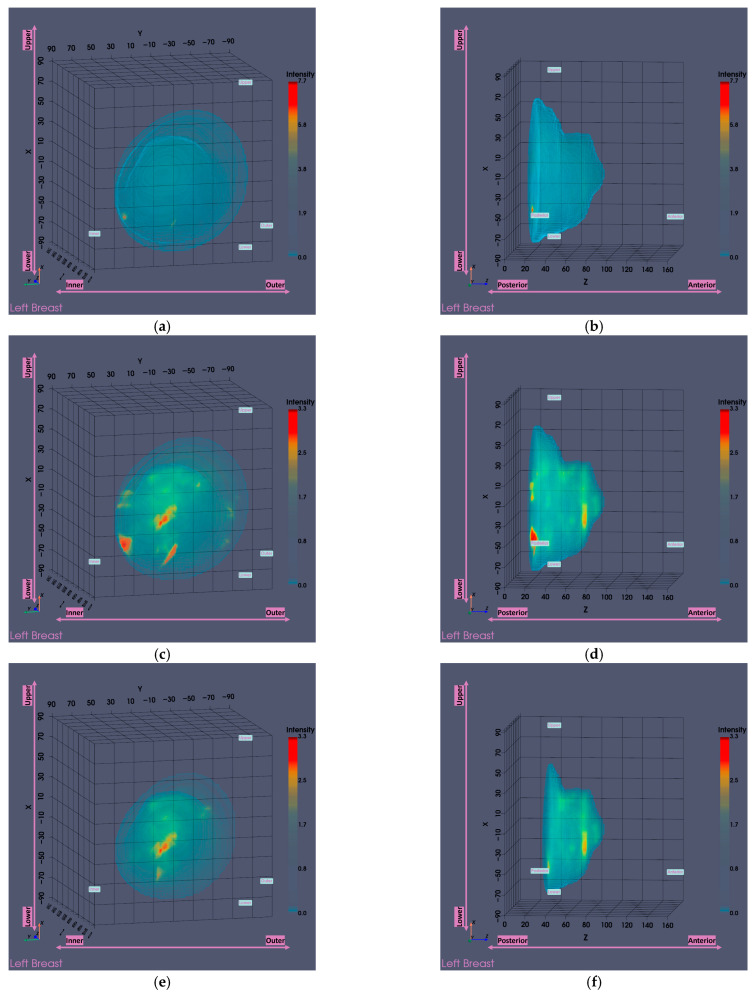

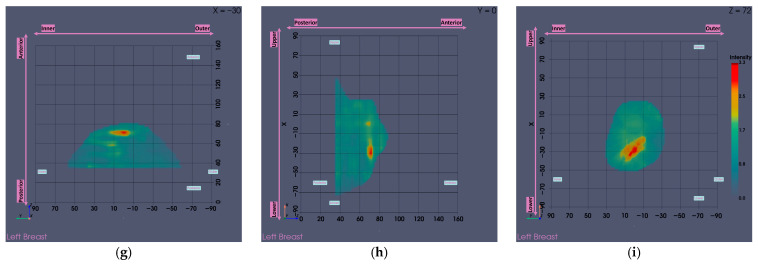
Visual comparison of Wavelia imaging results before and after the application of the tool for Patient 059, Left breast. The images correspond to: (**a**) Full scan default view; (**b**) Full scan lateral view; (**c**) Full scan default view with intensity saturation; (**d**) Full scan lateral view with intensity saturation; (**e**) Partial scan default view; (**f**) Partial scan lateral view; (**g**) x-cut at the centroid of the extracted ROI; (**h**) y-cut at the centroid of the extracted ROI; (**i**) z-cut at the centroid of the extracted ROI.

Figure 14 presents the Wavelia MWBI imaging results for Patient 059, Left breast, with and without the application of the partial scan generation tools. This representative case illustrates the impact of the tools in addressing the common issue of the ROI appearing ambiguous and non-dominant in the full 3D MWBI scan, due to inaccurate breast surface estimation near the chest wall, leading to strong imaging artifacts in that area.

As illustrated in Figure 14, inaccurate breast surface estimation near the chest wall leads to an unrealistic reconstructed envelope, where regions that should be identified as transition liquid are instead considered as interior breast tissue. This misrepresentation introduces a high-intensity artificial response in that area, clearly visible in Figure 14a–d, which dominates the imaging scene both in size and intensity, making it challenging to clearly distinguish and accurately extract the ROI associated with the breast lesion.

After evaluating the partial scan generation tools (with both tools triggered and the *area-breakpoint* criterion applied), a significant portion of the upper breast region is excluded, effectively removing the low-fidelity heights that contained the prominent artifacts. As a result, the actual region of interest previously obscured by the artificial high-intensity response near the chest wall becomes clearly visible, as illustrated in Figure 14e,f, where the artifact has been successfully eliminated and the target is now distinctly identifiable. To ensure a fair visual comparison, an intensity-saturated version of the full scan output is also shown in Figure 14c,d, using the same intensity range as that of the partial scan.

**Figure 15:**
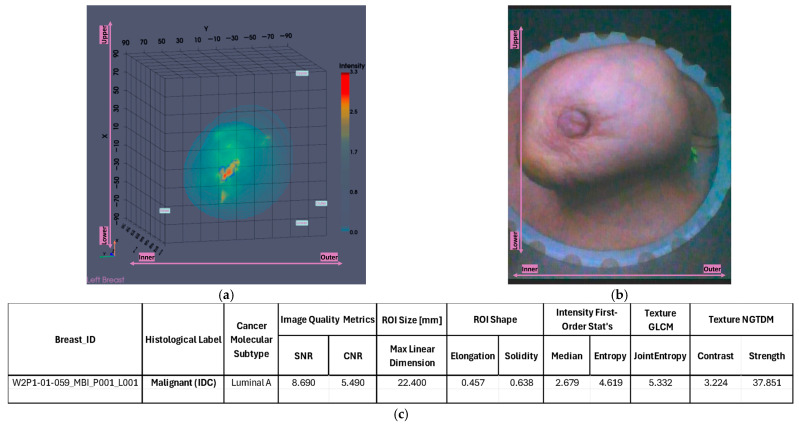
Patient 059, Left breast scan, Invasive Ductal Carcinoma: Illustrative subset of the Wavelia MWBI image analysis outputs. Packaged reporting per extracted ROI for clinical analysis. The images correspond to: (**a**) Partial scan default view with extracted ROI superimposed in blue; (**b**) Endoscopic camera view; (**c**) Main ROI features computations.

The output slices at the ROI location in Figure 14g–i provide a clearer view of the region of interest. The extracted ROI, now clearly spotted, is shown Figure 15 for further sizing and characterization.

The extracted ROI closely matches the lesion location as depicted in the related reference modalities previously presented in Figure 11, showcasing its accurate localization. Moreover, its dimension is calculated to be 22.4 mm as shown in Figure 15c, which is also very similar to the size computed based on the US (20 mm), suggesting the potential of Wavelia for precise lesion sizing.

For a more complete presentation of the Wavelia outputs, the low and high PC-FIB ranges’ partial scan outputs are presented next. The PC-FIB parameter, previously defined in [29,31,33], represents an assumed percentage of fibroglandular tissue involved along the electromagnetic waves' propagation path within the breast. The low PC-FIB range corresponds to values below 50% (lower breast density), while the high PC-FIB range includes values above 50% (higher breast density).

**Figure 16:**
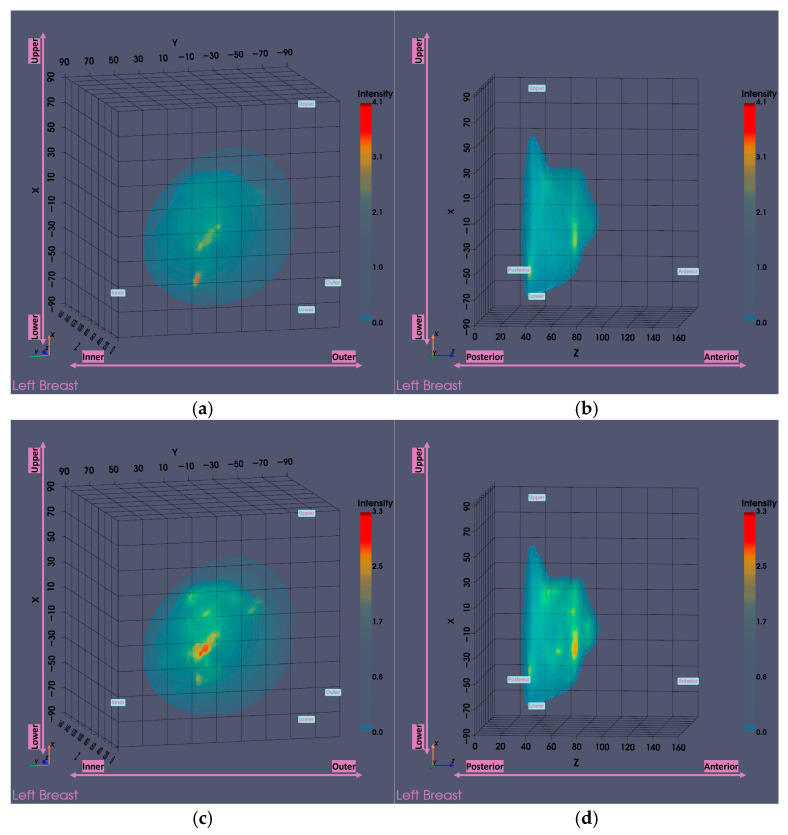
Visual comparison of Wavelia imaging results on low and high PC-FIB ranges for Patient 059, Left breast. The images correspond to: (**a**) Low PC-FIB default view; (**b**) Low PC-FIB lateral view; (**c**) High PC-FIB default view; (**d**) High PC-FIB lateral view.

It is noted that the low PC-FIB output shown in Figure 16a,b is inferior to the high PC-FIB one shown in Figure 16c,d in terms of ROI visibility, as a ghost target is highlighted in the posterior part of the reconstructed breast image due to non-realistic PC-FIB assumptions. The presented results indicate that better breast interior imaging is achieved by using high PC-FIB assumptions to model and approximate the speed of the electromagnetic waves when propagating through the nipple and retro-areolar zone of the breast, which is known to be associated with high density and increased permittivity values. In the specific case, given that the global averaged image is of similar quality, it was still the one retained for ROI extraction and feature analysis at the study group level. In the full analysis set, there was a total of 10 out of the 124 breasts (8.1%; 95% CI: [4.4–14.2%]) for which the high PC-FIB image was of clearly superior quality and selected for ROI extraction and characterization. The low PC-FIB image was selected in 8 out of the 124 (6.5%; 95% CI: [3.3–12.2%]) analyzed breasts instead.

### 3.2. Functional Analysis of the Partial Scan Generation Tools

This section evaluates the performance and impact of the developed tools when applied to cases involving relatively large and small breast sizes. Section 3.2.1 addresses cases involving larger breasts, highlighting both the overall impact of the corresponding tool on the affected cases and specific examples that illustrate its functionality. Section 3.2.2 follows with an equivalent analysis for the tool designed for smaller breasts, adopting the same structure as Section 3.2.1.

#### 3.2.1. Large Breasts Tool

**Figure 17:**
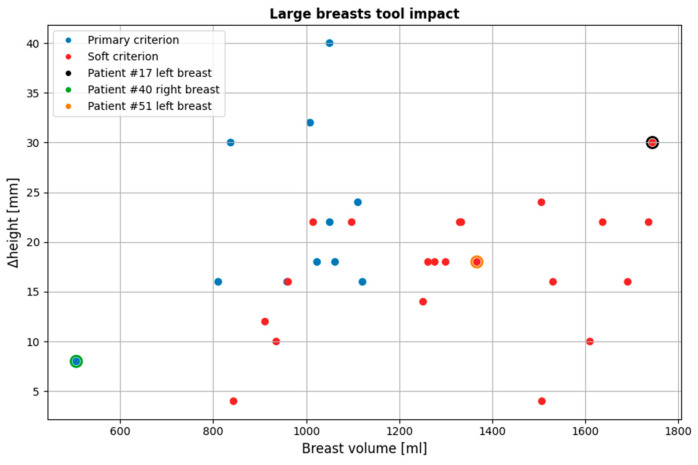
Overview of all cases affected by the large breasts tool. The three highlighted points indicated by black, green and orange outlines represent specific examples that illustrate the tool’s functionality, which are further detailed in this section. The y-axis (Δ*height*) indicates the vertical extent [mm] of the pendulous breast’s MWBI scan that is rejected due to insufficient quality.

Figure 17 illustrates the impact of the large breasts tool on the currently available dataset. The tool affected 32 of the 124 breasts (25.8%; 95% CI: [18.9–34.2%]), for which the rejected portion of the MWBI scan averaged 18.75 mm (SD = 7.68 mm; 95% CI: [15.98–21.52 mm]). Binomial proportions are reported with 95% confidence intervals (CIs), calculated using the Wilson score method; similarly, mean values are reported with 95% CIs calculated using the Student’s *t*-distribution, assuming approximate normality of the data. The same methods apply to all subsequent proportion and mean estimates, respectively.

As it is observed, for larger volumes, the soft criterion is more dominant. This is because, as stated before in 2.5.1, very large breasts tend to be closer to the probe array for a greater portion of the breast compared to smaller ones. This highlights the importance of the soft criterion, as even in such cases where it is applied, the portion of the breast rejected is not negligible. By solely relying on the primary criterion, the resulting partial scan would be significantly limited, leading to reduced diagnostic value.

It is worth noting that certain smaller breasts are affected by this tool as well. The underlying reason for this, as previously discussed, is that smaller breasts are placed deeper in the scanner, occasionally causing excess non-breast tissue to enter the scanner. This can lead to inaccurate breast contour estimation in the near-chest wall region, where the contour may appear significantly wider than its actual dimensions, thereby triggering the tool.

**Figure 18:**
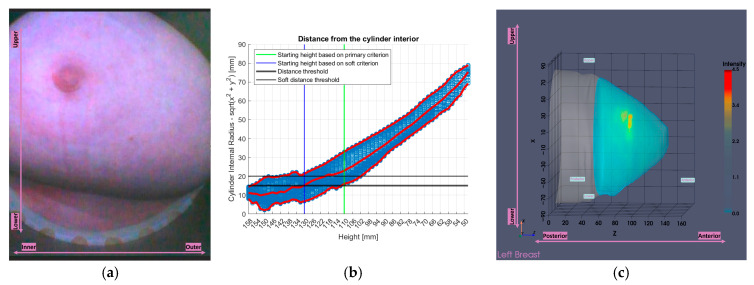

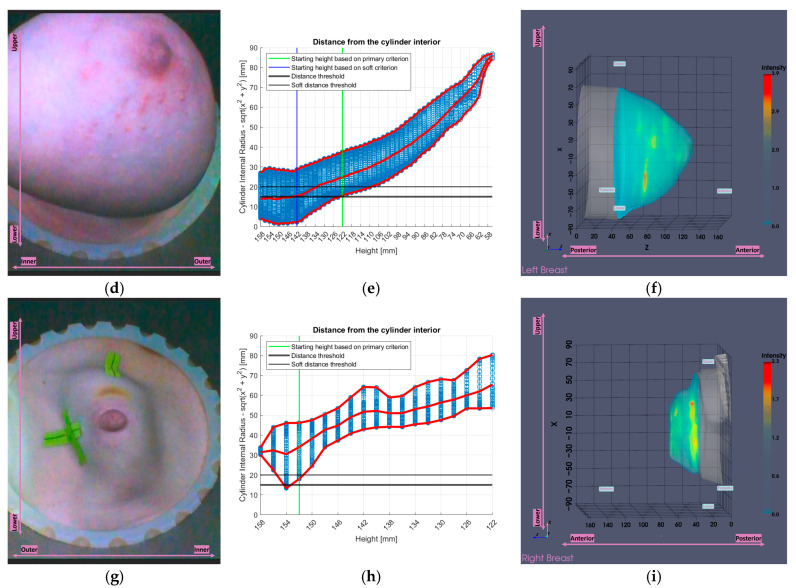
Visual examples illustrating the tool’s application on three cases. In the third column, the semi-transparent gray region indicates the area marked for exclusion by the tool. The images correspond to: (**a**) Endoscopic camera view of Patient 017, Left breast; (**b**) Tool analysis for Patient 017, Left breast; (**c**) Wavelia imaging results for Patient 017, Left breast; (**d**) Endoscopic camera view of Patient 051, Left breast; (**e**) Tool analysis for Patient 051, Left breast; (**f**) Wavelia imaging results for Patient 051, Left breast; (**g**) Endoscopic camera view of Patient 040, Right breast; (**h**) Tool analysis for Patient 040, Right breast; (**i**) Wavelia imaging results for Patient 040, Right breast.

Three visual examples are presented: two representative cases for which the tool was developed (large breasts), and one case (the leftmost point of the scatter plot of Figure 17 representing a small breast) where the tool was initially not intended to be triggered. These examples are shown in Figure 18.

The first two patients in Figure 18a–f are typical examples for which the tool was designed. The large volumes are clearly visible in the endoscopic views in Figure 18a,d and are also mirrored in the tool’s analysis illustrations in Figure 18b,e, which show numerous height values and generally low minimum values of distance between the breast and the probe array, compared to the third patient in Figure 18g–i. In the Wavelia imaging results in Figure 18c,f, the full estimated surfaces of the breasts are accurately defined; however, the filtered area is significant. This emphasizes the importance of the soft criterion. Without it, the primary criterion alone would have excluded even more of the breast region, potentially leading to outputs of limited clinical relevance.

Regarding the last patient in Figure 18g–i, the endoscopic view in Figure 18g shows that chest wall components have entered the scanner. This leads to inaccurate contour estimation, resulting in a reconstructed surface shown in Figure 18i that appears wider than the actual boundary defining the interior of the patient’s breast. This artificial widening is reflected in Figure 18h, which causes the tool to be triggered.

#### 3.2.2. Small Breasts Tool

**Figure 19:**
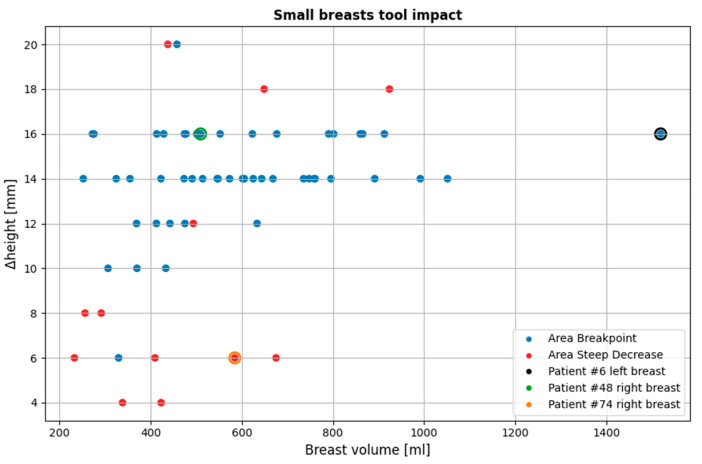
Overview of all cases affected by the small breasts tool. The three highlighted points indicated by black, green, and orange outlines represent specific examples that illustrate the tool’s functionality, which are further detailed in this section. The y-axis (Δ*height*) indicates the vertical extent [mm] of the pendulous breast’s MWBI scan that is rejected due to insufficient quality.

Figure 19 illustrates the impact of the small breasts tool on the currently available dataset. The tool affected 64 of the 124 breasts (51.6%; 95% CI: [42.9–60.2%]), with an average rejected MWBI scan portion of 13.38 mm (SD = 3.60 mm; 95% CI: [12.48–14.27 mm]).

As is observed, the *area-breakpoint* criterion is significantly more dominant, as it concerns 52 of the 64 breasts (81.3%; 95% CI: [70.0–88.9%]). This is due to the fact that the phenomenon of relatively equal contour areas near the chest resulting in almost flat regions in the piecewise linearly approximated ***areas*** graph, followed by a consistent contour area decrease, is common. It is also worth noting that for most cases where the *area-breakpoint* criterion was applied, the rejected portion of the MWBI scan averaged 14.23 mm (SD = 2.16 mm; 95% CI: [13.63–14.83 mm]). Both the narrow CI and the scatter plot in Figure 19 support a consistent trend in the imaging setup, which warrants further investigation.

As revealed in Figure 19, there are specific cases that triggered the tool, which are not classified as small breasts in terms of volume. Nearly all of them are affected by the *area-breakpoint* indicator, pointing out the systematic nature of the aforementioned phenomenon, which risks being associated with the MWBI scanner itself and not with the breast geometry.

**Figure 20:**
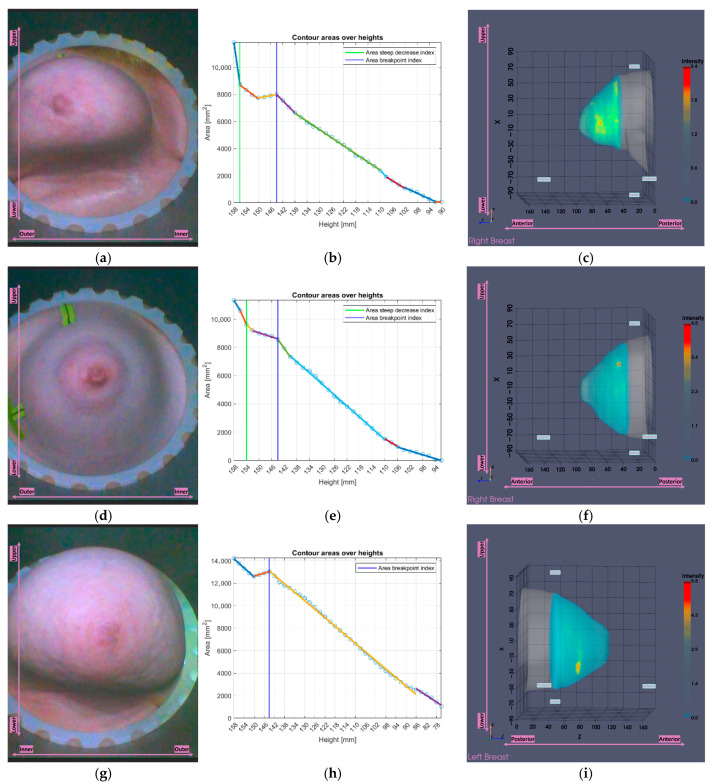
Visual examples illustrating the tool’s application on three cases. In the third column, the semi-transparent gray region indicates the area marked for exclusion by the tool. The images correspond to: (**a**) Endoscopic camera view of Patient 048, Right breast; (**b**) Tool analysis for Patient 048, Right breast; (**c**) Wavelia imaging results for Patient 048, Right breast; (**d**) Endoscopic camera view of Patient 074, Right breast; (**e**) Tool analysis for Patient, 074 Right breast; (**f**) Wavelia imaging results for Patient 074, Right breast; (**g**) Endoscopic camera view of Patient 006, Left breast; (**h**) Tool analysis for Patient 006, Left breast; (**i**) Wavelia imaging results for Patient 006, Left breast.

Figure 20 presents three visual examples where the small breasts tool output was selected, two nominal use-cases for which the tool algorithmics were actually designed (small breasts), and one case (rightmost point of the scatter plot of Figure 20, representing a large breast) where the tool was unexpectedly triggered.

In the tool’s analysis illustrations for all three cases in Figure 20b,e,h, a nearly flat region is observed near the upper height levels, followed by a clear downward trend. These examples include two small breasts shown in Figure 20a–f, for which the tool was designed, and one large breast shown in Figure 20g–i, where the tool was not intended to be activated, yet the same pattern is consistently present, triggering the *area-breakpoint* criterion and illustrating its systematic nature, as previously discussed. The first two small breasts are typical examples where excess chest tissue enters the scanner, resulting in rapid area decrease at upper heights and consequently triggering the *area–steep-decrease* criterion.

### 3.3. Breast Volume-Based Evaluation of the Joint Impact of the Partial Scan Generation Tools

**Figure 21:**
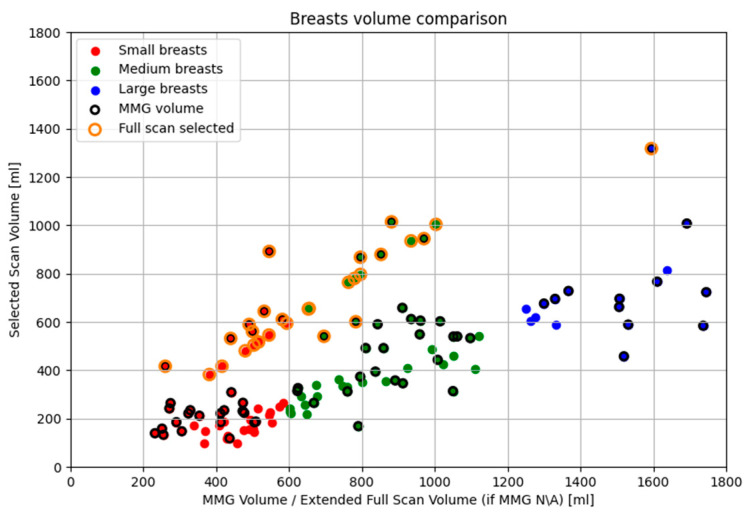
Scatter plot of cases affected by both partial scan generation tools. Data points depict the relationship between the full and the selected breast volume for the 124 breasts.

In this final section, the overall performance of the partial scan generation tools is presented in terms of volume reduction based on Figure 21.

In the Wavelia#2 MWBI clinical investigation, the breast volumes are categorized and analyzed into three groups: small (<600 mL), medium (600–1200 mL) and large (>1200 mL). Volume estimation is based on MMG-derived data when ‘for processing’ DICOM files were available from the hospital, enabling analysis using the Volpara Lab software for both volume and volumetric breast density. This applied to 67 out of 124 breasts (54.03%). Although the total number of patients with available mammographic data was significantly higher (53 out of 62 evaluable patients, 85.48%), only this subset had suitable MMG data in the required format for processing with Volpara Lab software. For the remaining 57 breasts (45.97%), volume estimates were derived from Wavelia MWBI scans.

The partial scan generation tools' outputs were selected in 96 of the 124 breasts (77.4%; 95% CI: [69.3–83.9%]), while the full scan was used for the remaining 28 breasts. Among these 28 scans, 24 also had available partial scans; however, the full scan was retained to preserve lesions in the otherwise excluded regions, as their positions were known from reference imaging modalities (MMG, US, or MRI). These scans represent 24 of the 120 (20.0%; 95% CI: [13.8–28.0%]) of the cases where either tool was triggered.

In the scatter plot of Figure 21, most full scan cases lie along the *y* = *x* line, as expected. Those slightly deviating from it correspond to breasts where volume was MMG-based (points with black and orange borders), inherently differing from Wavelia MWBI-derived estimates. Points below the line indicate underestimation by Wavelia compared to MMG, while those above show mild overestimation. As previously discussed in Section 2.5.2, small breasts tend to be immersed deeper into the scanner, leading to excess tissue also entering the scanner and consequently inflating the Wavelia MWBI-based estimate of the scanned breast volume. This explains why most of the points above the *y* = *x* line correspond to small breasts, confirming this tendency.

Regarding small breasts where the partial scan generation tool output was selected, particularly those near the upper threshold of 600 mL, it is observed that the selected volumes are significantly smaller than the corresponding full volumes. As explained in Section 2.5.2, in cases of smaller breasts, it is frequent that extramammary tissue or other anatomical structures near the chest wall enter the scanner, causing an artificial widening of the reconstructed surface. The tool aims to identify and exclude these regions. When applied successfully, this part of the breast is discarded, resulting in a serious reduction in the estimated breast volume, as this excluded region constitutes a notable fraction of the total mass.

Concerning large breasts for which the partial scan generation tool was triggered, a similar fact is observed. The selected volumes are substantially smaller than the full volumes, sometimes less than half. As described in Section 2.5.1, large breasts have extended peripheries, particularly near the chest wall, which can be further distorted by the buoyant force of the immersion liquid. This distortion brings the skin closer to the probe array, causing the tool to be triggered, filtering the upper portion of the breast. Since the breast periphery is reduced towards the nipple, a significant portion of the breast volume is concentrated in the upper heights. Consequently, the filtered regions often correspond to substantial portions of actual breast tissue that cannot be scanned with sufficient quality. As a result, these regions are excluded to preserve imaging fidelity. Wavelia MWBI therefore outputs partial breast images in such cases, ensuring that the retained data remains reliable for radiological interpretation and clinical analysis. This effect is even more noticeable in very large breasts, where the skin is naturally close to the array across many height levels.

Finally, for the medium-sized breasts where either partial scan generation tool was applied, the breast volume changes follow one of the previously mentioned trends. Breasts with volumes near the lower end of the medium range (600 mL) tend to behave similarly to small breasts, while those towards the upper end (1200 mL) exhibit characteristics closer to large breasts, resulting in comparable tool behavior.

## 4. Discussion

A set of new modules tailored to the Wavelia#2 MWBI design to allow optimization of the breast insertion in the scanner was found to have better control on the breast centering and orientation in the scanner, and proper definition of the portion of the vertical MWBI scan of sufficient quality, and were presented in this article. While maintaining out of the scope of this paper the core Wavelia MWBI imaging methodology, which was upgraded for the Wavelia#2 system as defined in [32], the focus of the presented study is on the importance of proper management of the geometry and deformability of the breast under scan, to assure quality of scan and a valid reference reporting space for the MWBI imaging outputs.

A significant portion of the paper is dedicated to the newly developed methodology for systematic and automated definition of partial MWBI scan, which was introduced for the first time in the Wavelia#2 clinical investigation. The new module allows more reliable reporting of MWBI imaging and image analysis outputs in a restricted domain, after excluding a portion of the scan close to the examination table, potentially associated with imaging artefacts/ghost ROIs of strong intensity. The rationalized definition of partial scan of high fidelity, in cases of very large breasts, or in cases of breasts being too small such that part of the chest and pectoral muscle enters the MWBI scanner and dominates the 3D MWBI image intensity, was presented in detail, being organized in a bi-modal configuration: large breasts mode and small breasts mode.

It was demonstrated that the application of the new module to the full analysis set of the Wavelia#2 clinical investigation was automatically enabled in the majority (77.4%; 95% CI: [69.3–83.9%]) of MWBI breast scans and resulted in exclusion of a non-negligible portion of the MWBI scans from the image formation and analysis process. This contributed to: (a) elimination of a non-negligible amount of unspecified ROIs, which would otherwise deteriorate drastically the result of the preliminary qualitative assessment of specificity of the Wavelia#2 MWBI device, as reported in [51] and discussed in Section 2.2.2; (b) improved the achieved Contrast-to-Noise Ratio (CNR) of the clinically relevant ROIs, contributing to a clearer depiction and a more accurate delineation and texture pattern retrieval, thus contributing positively to the statistically significant diagnostic accuracy achieved for a set of malignant-to-benign lesion discriminating ROI features in this study [51]. Considering the notable deterioration of the achieved performance in case of overall full breast scan analysis, which triggered the design of the partial scan definition tool in the early phase of the data collection for this study, systematic evaluation of the full data processing pipeline, up to ROI extraction, analysis and reporting, was not considered worthwhile to be included in the study final analysis and is not reportable for all the full breast scans. The earlier FiH study with the Wavelia#1 MWBI device was limited to analysis of a single ROI per patient examination dataset, thus concentrating on initial technological feasibility and detectability of the dominant discrete lesion of each patient, while neglecting any notion of specificity assessment for the MWBI modality. A meaningful MWBI dataset for such an assessment was for the first time acquired in the Wavelia#2 pilot clinical investigation, with the contribution of the technical modules presented in this article being critιcal.

In the case of very small breasts, which were deeply inserted in the Wavelia#2 MWBI scanner, the partial scan definition consisted of intended isolation of the pendulous breast from the pectoral muscle and chest wall being inserted in the scanner in an uncontrolled manner in the current implementation. In optimal operation conditions, the reduced scan should not be associated with any loss of information in such cases and rather functions as a numerical spatial filter allowing to mitigate ghost ROIs and maintain the specificity of the MWBI imaging modality to a good level.

In the case of large breasts not fully inserted in the scanner, and/or inserted in too close vicinity to the probe array, thus generating inter-probe coupling-related interference of uncontrolled patterns and strong intensity levels, the reduced scan defines an actual partial scan of these breasts, where acceptable quality can be assured. As reported in Figure 21, even though very large breast volumes up to 1800 mL were accepted for inclusion in this study, the MWBI imaging and image analysis reported in such cases concern partial scans of these breasts. The maximal breast volume for which MWBI imaging results were reported in this study was approximately 1000 mL, with a single case of ~1300 mL breast being maintained for imaging and reporting in the full scanned volume. In such cases, lesions might have been removed if present within the portion of the full breast volume being excluded from imaging and analysis due to insufficient quality of the MWBI scan. As earlier mentioned, in the scope of the current clinical investigation, a full breast scan was forced to be retained to avoid cutting out of known lesions, as per available standard-of-care reference imaging. Going beyond this clinical investigation, a partial MWBI image of the breast would be only available in such cases, actually limiting the maximal size of breast that can be accommodated and fully handled by each version of the MWBI scanner.

## Data Availability

The data are not publicly available due to Institutional (University Hospital of Galway, Ireland) regulations and non-violation of patients’ privacy.

## Appendix A. Large Breasts Tool: Technical Specifications

Let $S_{h}$ be the set of the *n_h_* breast surface heights. For each height $h_{i}\in S_{h}$, the set of the $n_{h_{i}}$ contour points is denoted as $S_{p,h_{i}}$, with coordinates $\left(x_{h_{i}},\;y_{h_{i}}\right)$. The Euclidean distance from each point *p* to the probe array’s internal radius *R* is calculated as $d_{h_{i},\;p}=R-\sqrt{{x(p)}_{h_{i}}^{2}+{y(p)}_{h_{i}}^{2}}$. Heights where more than *perc* = 5/360 (i.e., a 5° arc) of contour points lie within a threshold distance *thres_global_* = 15 mm from the probe array are collected into a set *S_h,filt_*. The values of *thres_global_* and *perc* have been set empirically.

(A1) $$S_{h,filt}=\left\{h_{i}\in S_{h}\;\right|\sum_{p=1}^{n_{h_{i}}}1_{d_{h_{i},\;p}\leq {thres}_{global}}\geq perc\cdot n_{h_{i}}\}$$

The top height of the partial scan, *h_partial_*, is set as the maximum height in *S_h_* below the minimum height in *S_h,filt_*, ensuring sufficient distance from the probe. For very large breasts, this approach may exclude a substantial portion of the scan. To balance measurement fidelity with adequate breast coverage, a softer version of the tool is also applied. This defines two new filtered height sets *S_h,filt,soft,k_, k =* 1, 2, as follows.

(A2) $$S_{h,filt,soft,k}=\left\{h_{i}\in S_{h}\;\right|\;mean(d_{h_{i}})\leq t_{k}\},\;k=1,\;2,\;t_{k}=\left\{\begin{array}{c} {thres}_{global},\;k=1\\ {thres}_{soft},\;k=2 \end{array}\right.$$

The value of *thres_soft_* is also empirically set to 20 mm. Two candidate soft top heights *h_partial,soft,k_*, *k* = 1, 2 are computed similarly to *h_partial_*. The final soft top height *h_partial,soft_* is subsequently computed, considering *h_uppermost_*, the device’s uppermost scanning position.

(A3) $$h_{partial,soft}=\left\{\begin{array}{c} h_{partial,soft,1},\;{val}_{decision}>0\\ h_{partial,soft,2},\;{val}_{decision}\leq 0 \end{array}\right.\;{val}_{decision}=\left|h_{uppermost}-h_{partial,soft,1}\right|-\left|h_{partial,soft,2}-h_{partial}\right|$$

The intuition behind the branched decision logic is to ensure the soft output remains sufficiently distinct from both the full scan and the primary partial scan, which is considered too restrictive for the given breast.

Table A1 summarizes the key parameters and variables used in the large breast tool, including their symbols, values, units and a short description.

**Table A1:** Key parameters and variables for the large breasts tool.

Symbol	Value (Units)	Description
$S_{h}$	— (—)	Set of all measured heights along z-axis
$n_{h}$	— (—)	$\mathrm{Number}\;\mathrm{of}\;\mathrm{heights}\;\mathrm{in}\;S_{h}$
$h_{i}$	— (mm)	$\mathrm{Variable}\;\mathrm{for}\;\mathrm{referring}\;\mathrm{to}\;\mathrm{heights}\;\mathrm{in}\;S_{h}$
$S_{p,\;h_{i}}$	— (—)	$\mathrm{Set}\;\mathrm{of}\;\mathrm{contour}\;\mathrm{points}\;\mathrm{at}\;\mathrm{height}\;h_{i}$
$\left(x_{h_{i}},\;y_{h_{i}}\right)$	— (mm)	$\mathrm{Cartesian}\;\mathrm{coordinates}\;\mathrm{of}\;\mathrm{contour}\;\mathrm{points}\;\mathrm{at}\;\mathrm{heights}\;h_{i}$
$n_{h_{i}}$	— (—)	$\mathrm{Number}\;\mathrm{of}\;\mathrm{contour}\;\mathrm{points}\;\mathrm{at}\;\mathrm{height}\;h_{i}$
R	90 (mm)	Probe array internal radius
p	— (—)	Variable for referring to contour points
$d_{h_{i},p}$	— (mm)	Euclidean distance from contour point p$\;\mathrm{at}\;\mathrm{height}\;h_{i}$ to probe the array
perc	5/360 (—)	Primary indicator percentage threshold of contour points close to the probe array
${thres}_{global}$	15 (mm)	Primary indicator distance threshold
$S_{h,\;filt}$	— (—)	Set of heights marked for filtering by primary indicator
$h_{partial}$	— (mm)	Maximum preserved height by primary indicator
$S_{h,\;filt,\;soft,\;k}$	— (—)	Candidate sets of heights marked for filtering by soft indicator
${thres}_{soft}$	20 (mm)	Soft indicator supplementary distance threshold
$h_{partial,\;soft,\;k}$	— (mm)	Candidate maximum preserved heights by soft indicator
$h_{partial,\;soft}$	— (mm)	Selected maximum preserved height by soft indicator
$h_{uppermost}$	158 (mm)	Maximum scanning position of the MWBI scanner

## Appendix B. Small Breasts Tool: Technical Specifications

To identify near-chest wall regions where geometry is notably disrupted by non-mammary tissue, we define the ***areas*** vector, containing the contour areas at the given heights *h_i_*∈*S_h_*, starting from *h_uppermost_* down to the nipple region. The contour areas are the areas in mm^2^ contained within the boundaries of the breast contours at different heights. To robustly analyze this profile, the ***areas*** vector is approximated by a piecewise linear representation, upon which two indicators are computed. The overall procedure consists of three main phases: (i) segmentation, where the ***areas*** vector is iteratively divided into linear sub-segments, (ii) post-processing (merging), where short segments are combined to enhance structural coherence of the piecewise approximation, (iii) indicators computation, where the two indicators are derived from the final ***areas*** vector approximation.

The initial piecewise linear segmentation of the ***areas*** vector is performed iteratively to approximate the breast contour areas while preserving structural fidelity. The procedure is as follows:

1. Initialization: Treat the full ***areas*** vector as a single segment.
2. Select segment: Identify the segment *seg* (containing *n* elements) with the largest deviation from linearity, quantified by its Mean Squared Error (MSE).
3. Evaluate candidate splits: For each potential breakpoint *b* within *seg*:
  - Divide *seg* into two sub-segments:
    ${seg}_{1}=seg(1:b)$ and ${seg}_{2}=seg(b+1:n)$.
  - Fit a linear model to each sub-segment and compute the corresponding MSEs:
    (A4) $${MSE}_{1}\left(b\right)=\frac{1}{b}\cdot \sum_{k=1}^{b}{\left(seg(k)-\hat{{seg}_{1}}(k)\right)}^{2},\;{MSE}_{2}\left(b\right)=\frac{1}{n-b}\cdot \sum_{k=b+1}^{n}{\left(seg(k)-\hat{{seg}_{2}}(k-b)\right)}^{2}$$
  - Compute the joint MSE (*MSE_joint_*) for the split: ${MSE}_{joint}={MSE}_{1}+{MSE}_{2}$.
4. Select optimal split: Choose the candidate breakpoint that minimizes *MSE_joint_* and update the segments list.
5. Iterate: Repeat steps 2–4, always selecting the segment with the highest MSE, until the longest segment remains unchanged between two consecutive iterations. This indicates that further splits would not meaningfully reduce the total MSE (*MSE_total_*) of the approximation, which is defined as the sum of the MSEs of all current segments.

This iterative segmentation ensures that each sub-segment is well-approximated by a linear fit, avoiding over-segmentation caused by minor fluctuations in the ***areas*** vector.

Following the initial piecewise linear segmentation, a post-processing step is applied to iteratively merge any two-point segments commonly produced during the previous step, in order to enhance structural coherence and facilitate subsequent computations. The procedure is as follows:

1. Identify candidates: Locate all two-point segments in the current segments list.
2. Evaluate potential merges: Merge each candidate individually with each of its neighboring segments. Fit a new linear model to the merged data and compute its error *MSE_merged_*. The error increase is quantified as $\Delta MSE={MSE}_{merged}-({MSE}_{1}+{MSE}_{2})$, where *MSE*_1_, *MSE*_2_ are the errors of the two original segments prior to merging.
3. Select merge: Apply the merge with the smallest Δ*MSE*, provided that the updated *MSE_total_* after merging remains below a reference threshold:
  (A5) $$\frac{{MSE}_{total}}{{MSE}_{ref}}\leq \tau$$
4. **Iterate**: Repeat steps 2,3 until no two-point segments remain or no candidate merge satisfies the MSE constraint.

The reference MSE (*MSE_ref_*) is the total MSE before the merging process begins and the threshold *τ* has been empirically set to 1.2, allowing for a maximum 20% total MSE increase to avoid significant degradation of the approximation quality.

After obtaining the piecewise linear approximation of the ***areas*** vector, two indicators are derived to identify near-chest wall regions affected by non-breast tissue: *area-breakpoint* and *area-steep-decrease*. The first one, *area-breakpoint*, captures structural transitions in the ***areas*** profile, specifically the transition from relatively flat regions potentially indicating non-mammary tissue, to monotonically decreasing regions corresponding to the expected breast envelope, where internal contour areas progressively decrease toward the nipple. This is achieved by analyzing slope changes between adjacent linear segments as follows:

1. Compute segment slopes: For each segment *i*, compute the slope *s_i_* of the linear fit.
2. Compute slope ratios: For each pair of adjacent segments [*i* − 1, *i*], calculate the slope ratio: r=si−1si
3. Flag potential breakpoints: A breakpoint is flagged if all the following conditions are met:
  - Current slope is negative (*s_i_* < 0).
  - Either the preceding slope is non-negative (*s_i-1_* ≥ 0) or *r* ≤ 0.5.
  - The three-segment region [*i* − 1, *i* + 1] contains at least *minEvaluableLength* points (empirically set to 5).
  - The region length is maximal among all evaluated regions.
4. Refine breakpoint location: Apply a two-segment linear approximation over the three-segments region [*i* − 1, *i* + 1] to determine the precise breakpoint.
5. Select final breakpoint: Accept the breakpoint only if it lies within the first third of the ***areas*** vector, ensuring it corresponds to the near-chest wall region.

This approach ensures the final breakpoint reflects a meaningful change in slope dynamics while remaining robust to minor fluctuations.

The second indicator, *area-steep-decrease*, detects sharp reductions in contour areas near the chest wall by identifying segments where the slope of the linear area approximation drops abruptly, indicating a structural transition caused by non-breast tissue entering the scanner. Unlike the *area-breakpoint* indicator, this criterion targets steep area reductions, offering a complementary assessment. The procedure is as follows:

1. Fit global slope: Fit a linear model to the entire ***areas*** vector to obtain the global slope *s_global_* (always negative).
2. Identify steep segments: Mark segments with slope *s_i_* ≤ 2·*s_global_* located within the first half of the ***areas*** vector, ensuring their relevance to the near-chest wall region.
3. Group adjacent steep segments: Combine consecutive steep segments into regions.
4. Select final region: Choose the longest region among all steep regions. The last index of this region defines the boundary of the non-breast tissue.

This approach robustly identifies near-chest wall regions with sharp area decreases, complementing the *area-breakpoint* indicator in detecting non-breast tissue regions entering the scanner.

Table A2 summarizes the key parameters and variables specific to the small breast tool, including their symbols, values, units and a brief description. Parameters and variables already defined in Appendix A are omitted for brevity.

**Table A2:** Key parameters and variables for the small breasts tool.

Symbol	Value (Units)	Description
areas	— (mm^2^)	Vector of breast contour areas at descending height order
${MSE}_{joint}$	— (mm^4^)	Sum of 2 segments MSEs for evaluating possible split
${MSE}_{total}$	— (mm^4^)	Sum of all current segments MSEs
${MSE}_{merged}$	— (mm^4^)	Segment MSE for evaluating possible merge
ΔMSE	— (mm^4^)	MSE difference for quantifying merge impact
${MSE}_{ref}$	— (mm^4^)	Total MSE before post-processing merging
τ	1.2 (—)	Maximum allowed increase in total MSE during post-processing merge
$s_{i}$	— (mm^2^/mm)	Variable for referring to the slope of the linear fit to segment i
r	— (—)	Ratio of segment i−1 slope to segment i slope
minEvaluableLength	5 (points)	Minimum number of points in a region to consider slope transitions valid for area−breakpoint
$s_{global}$	— (mm^2^/mm)	Slope of linear fit to full areas vector
